# Engineering Lipid Bilayer Membranes for Protein Studies

**DOI:** 10.3390/ijms141121561

**Published:** 2013-10-31

**Authors:** Muhammad Shuja Khan, Noura Sayed Dosoky, John Dalton Williams

**Affiliations:** 1 Electrical and Computer Engineering Department, University of Alabama in Huntsville, Huntsville, AL 35899, USA; E-Mail: msk0003@uah.edu; 2 Biological Sciences Department, University of Alabama in Huntsville, Huntsville, AL 35899, USA; E-Mail: nsd0003@uah.edu

**Keywords:** lipid bilayer membrane, nanoporous materials, silicon, transmembrane proteins, electrochemical impedance spectroscopy

## Abstract

Lipid membranes regulate the flow of nutrients and communication signaling between cells and protect the sub-cellular structures. Recent attempts to fabricate artificial systems using nanostructures that mimic the physiological properties of natural lipid bilayer membranes (LBM) fused with transmembrane proteins have helped demonstrate the importance of temperature, pH, ionic strength, adsorption behavior, conformational reorientation and surface density in cellular membranes which all affect the incorporation of proteins on solid surfaces. Much of this work is performed on artificial templates made of polymer sponges or porous materials based on alumina, mica, and porous silicon (PSi) surfaces. For example, porous silicon materials have high biocompatibility, biodegradability, and photoluminescence, which allow them to be used both as a support structure for lipid bilayers or a template to measure the electrochemical functionality of living cells grown over the surface as *in vivo*. The variety of these media, coupled with the complex physiological conditions present in living systems, warrant a summary and prospectus detailing which artificial systems provide the most promise for different biological conditions. This study summarizes the use of electrochemical impedance spectroscopy (EIS) data on artificial biological membranes that are closely matched with previously published biological systems using both black lipid membrane and patch clamp techniques.

## 1. Introduction

Membranes are ubiquitous in biological systems. They are selective barriers that maintain a chemical environment different from the surrounding medium. Biological membranes are crucial for cell protection, compartmentalization, signal transduction, and selective permeability; which allow specific molecules to be transported from and into the cell [1]. They are involved in various processes including enzymatic catalysis, molecular recognition, membrane fusion, cellular adhesion and many others. In this review, we will explore the lipid bilayer membrane (LBM), its structure, incorporation of proteins with LBMs, and the supporting surfaces used for probing different proteins the electrochemical impedance spectroscopy (EIS) to study the capacitance and resistance of a membrane, protein and fused protein in membrane.

### 1.1. Cell Membrane

**Figure 1:**
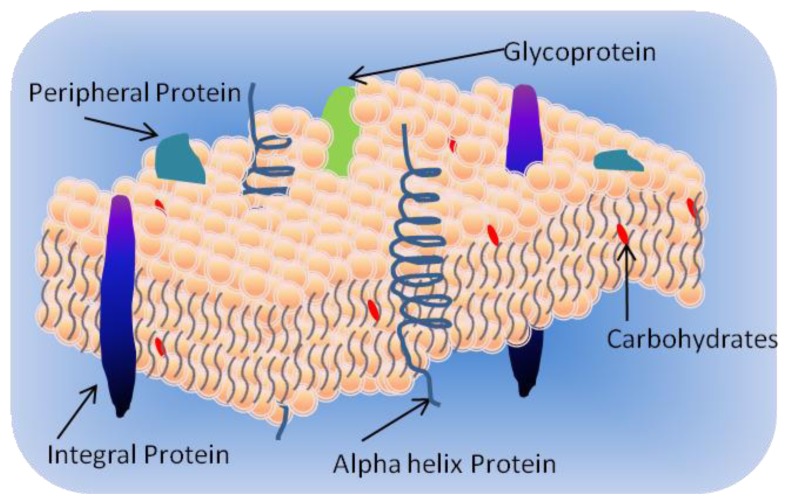
A lipid bilayer membrane (LBM).

Biological membranes display a very complex composition in terms of lipids and proteins. The cell membranes consist of three essential components: lipids, proteins and carbohydrates. The lipid components are mainly glycerophospholipids (also known as phospholipids), sphingolipids, and sterols. Phospholipids are amphipathic molecules composed of two basic parts, a polar head containing a phosphate group, and two non-polar fatty acid tails. The basic structure of a typical cell membrane is a bilayer of phospholipids and sphingolipids arranged in two layers with their polar heads to the outside, and their hydrophobic tails inside. This arrangement shelters the hydrophobic tails of the phospholipids from water and exposes only the hydrophilic heads to the water in the cytosol and extracellular fluid as shown in Figure 1. Inserted in the lipid bilayer are proteins responsible for providing channels and transporters for hydrophilic molecules and ions. These proteins include:

- Integral proteins which have one or more transmembrane domains;
- Peripheral proteins associated at the surface of the bilayer; and
- Lipid-anchored proteins covalently attached to the fatty acids in the membrane.

The selective permeability of these membranes is determined by the composition of their lipids, protein constituents, and carbohydrate components. Carbohydrates located just outside the bilayer are usually attached to the proteins or lipids forming glycoproteins and glycolipids, respectively [2,3]. In aqueous solutions, the amphiphilic single-tailed lipid molecules tend to form aggregates called micelles [4]. A micelle is a globular aggregate in which the hydrophobic tails are protected from water and only the polar heads are exposed to the surrounding aqueous medium. This arrangement prevents the unfavorable contact of hydrocarbons with water. Once the lipid molecules are put in water, the water molecules order themselves to form ice-berg-like cages around the hydrophobic groups of the lipid molecules in order to increase the entropy. Then to minimize the contact with water, the lipid molecules are forced closer to each other excluding the water molecules.

Coalescence of hydrophobic groups releases some of the ordered water, leading to a favorable increase in entropy.

The amphipathic nature of the membrane lipids helps to form the bilayer spontaneously in aqueous solutions. This occurs because the two tails of the membrane lipids give them a cylindrical shape. Thus, the minimum free energy in the system is obtained by creating a cylindrical wrap (or plane) instead of a spherical micelle. Furthermore, lipids tend to form a bimolecular sheet first to shield the non-polar tails from water. These bilayer leaflets can then fold easily to form a vesicle [5]. The final membrane structure is a flexible fluid comprising a mosaic of different lipids, proteins and carbohydrates. Molecules are not stationary, but constantly move laterally across the membrane structure. In 1972, scientists proved that each leaflet of bilayer is formed by a homogeneous environment of lipids in a fluid state including globular assembly of proteins and glycoprotein [6]. Further investigations [7] revealed that membrane lipids are organized into lateral micro-domains with a specific composition and a molecular dynamic that are different to those of the surrounding liquid crystalline phase. The lipid composition is different within two leaflets of the same membrane. Phosphatidylethanolamines and phosphatidylserines are mainly found in the inner leaflet of the plasma membrane, and phosphatidylcholines and sphingomyelines are essentially located in the outer leaflet. Cholesterols are equally distributed in the two leaflets of the lipid bilayer due to a fast flip-flop between the outer and the inner leaflets. Vesicle budding and membrane fusion are important biological processes, which result from the asymmetry across the membrane. Asymmetry is responsible for phase separation within one monolayer leaflet [8].

### 1.2. Classification of Membrane Lipids

Membrane lipids are classified as phospholipids, sphingolipids or sterols. Some of the membrane lipids are attached to carbohydrates and, therefore, are glycolipids. The attached carbohydrate moiety could be one to several molecules of galactose or glucose [5].

#### 1.2.1. Phospholipids

Two types of lipids comprise the majority of biological membranes, glycerophospholipids and sphingolipids. Glycerophospholipids consist of a core of sn-glycerol-3-phosphate that is esterified at its C1 and C2 positions to fatty acids (*i.e.*, the nonpolar aliphatic tails) and at its phosphoryl group to a group, X (*i.e.*, the polar head) [9]. The most common glycerophospholipids begin with the word “phosphatidyl” and end with serine, choline, ethanolamine, inositol, glycerol, *etc*., depending on the X-group attached to the phosphoryl group. In most cases, biomembranes have a high percentage of phospholipids compared to the other lipid components. For this reason the membrane is known as the phospholipid bilayer membrane.

#### 1.2.2. Sphingolipids

Sphingolipids are derivatives of the C18 amino alcohols, sphingosine, dihydrosphingosine, and their homologs. They include sphingomyelin, cerebrosides, and gangliosides. The formation of lateral microdomains, or rafts, within biological membranes is formed by interactions between sphingolipids and cholesterol [10]. Sphingomyelin contains a choline molecule attached to the hydroxyl group of ceramide.

#### 1.2.3. Sterols

Sterols are known to control biological processes and domain structure of cell membranes. The hydrophobic moiety of sterols is composed of polycyclic structures. Lanosterol is the sterol of prokaryotes and the chemical precursor of both cholesterol and ergosterol. Cholesterol is the most abundant sterol in eukaryotic plasma membranes, especially the erythrocyte membrane, as well as various sub-cellular compartments. In fungi, ergosterol is the most abundant. Plants are characterized by a more complex membrane sterol composition as they contain a mixture of stigmasterol, sitosterol, and two 24-ethyl sterols. Archebacteria and cyanobacteria contain hopanoids such as bacteriohopanetetrol [11].

The hydroxyl group in cholesterol is responsible for the amphiphilic nature of the molecule and consequently for its orientations in biological membranes. Cholesterol molecules orient themselves to the membrane bilayer with their hydroxyl group close to the polar head groups of adjacent phospholipid molecule. Due to the lipophilic nature of cholesterol, it plays a vital role in regulating the lipid membrane properties as it reduces permeability barriers, maintains membrane architecture, lipid organization and phase behavior and, thus, regulates membrane fluidity at different temperatures and affects membrane bending stiffness [12–15]. These temperature-dependent effects make cholesterol a “temperature buffer” for the lipid membrane. The ring structure of cholesterol is thought to reduce membrane fluidity at moderate temperatures by reducing phospholipid movement, while inhibiting solidification at low temperatures by disrupting the regular packing of phospholipids. It was also found to inhibit the formation of the interdigitated gel phase and to affect the crystalline lattice and mobility of the lipids. At high concentrations, cholesterols help in separating the phospholipids so that the fatty acid chains cannot move together and crystallize [16].

### 1.3. Membrane Proteins

**Figure 2:**
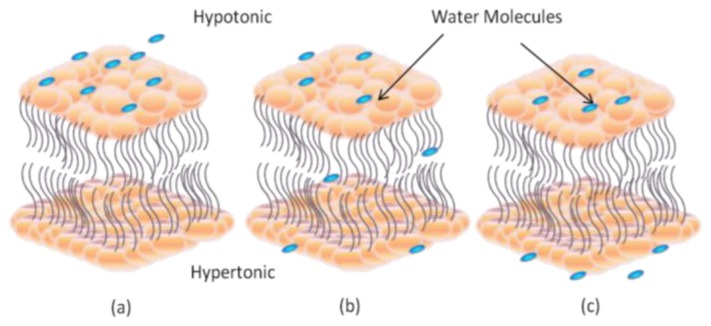
Bilayer lipid membrane osmosis process. (**a**) Water molecules flow at higher concentration (hypotonic); (**b**) Water molecules move from hypotonic region (high extracellular concentration) to hypertonic region (low extracellular concentration) across the LBM; (**c**) Water molecules are at equilibrium position.

Membrane contains two types of proteins: transmembrane and carrier proteins. Plasma membrane proteins perform several functions including transport, enzymatic activity, signal transduction, cell-cell recognition, intracellular joining, and attachment to the cytoskeleton and extracellular matrix. Transport is one of the most important functions of biomembranes. Depending on the energy requirements, there are two modes of membrane transport: passive and active. Passive transport is governed by diffusion. If the target molecules are moving according to their concentration gradient in the absence of other forces, the diffusion process will happen spontaneously and the molecules will be transported passively. Osmosis is a specific type of diffusion in which water molecules diffuse across the membrane down their concentration gradient. There are two important terms associated with this process: hypotonic and hypertonic. Water molecules flow from hypotonic (hyposmotic) environment towards the hypertonic (hyperosmotic) environment as shown in Figure 2.

Specific ions and a variety of polar molecules can easily be transported through the cell membranes. Hydrophilic substances pass through transport proteins found in the membrane in order to avoid contact with the non-polar lipid bilayer. This process is a more advanced form of passive transport known as facilitated diffusion or protein-aided passive transport. A channel protein is a type of transport protein that contains a hydrophilic channel through which certain molecules or ions can get from one side of the membrane to the other. Channel proteins that transport ions are known as ion channels. They are often gated channels, which open or close when stimulated. Another class of transport proteins is the carrier protein, which holds the cargo molecules and changes its shape in a way that delivers them across the membrane. Similar to ion channels, carrier proteins help in facilitated diffusion of molecules down their concentration gradient by passive transport [17].

Unlike passive transport, active transport requires energy to pump the target molecules against their concentration gradient, *i.e.*, from lower to higher concentration. This energy is usually provided through ATP hydrolysis. The transport proteins involved in active transport are all carrier proteins. One of the most well-known types of active transport is the Na^+^/K^+^ pump through which Na^+^ moves to outside the cell and K^+^ enters the cell. In order for this movement to happen, ATP must be consumed. One ATP molecule moves three Na^+^ ions and two K^+^ ions.

For transport on a large scale, there is endocytosis and exocytosis, that move large molecules or whole organisms inside and outside the cell, respectively [1,18]. Co-transport is the coupled passage of two materials across the cell membrane. When protons are actively transported across the membrane against the concentration gradient with the help of ATP, then it re-opens the door for another material (food), as well as moving back inside the cell membrane. Endocytosis is the import of materials to the cell by invagination of the plasma membrane. There are three types of endocytosis: phagocytosis, pinocytosis and receptor-mediated. In phagocytosis, pseudopods (extension of cell membrane) surround the item and form a vesicle to take it inside the cell across membrane. In pinocytosis, a fluid-filled vesicle of fluid is formed inside the membrane without the formation of pseudopods.

Some membrane proteins function as receptors with a binding site for a specific chemical messenger, such as a hormone. In some cases, binding to the external signaling molecule may induce the receptor protein to change its shape in order to relay the signal to the cytosol. In some other cases, the signaling molecule itself can pass through a protein channel and reach the cytoplasm [19].

## 2. Dynamic Structure and Formation of Lipid Bilayer Membrane (LBM)

**Figure 3:**
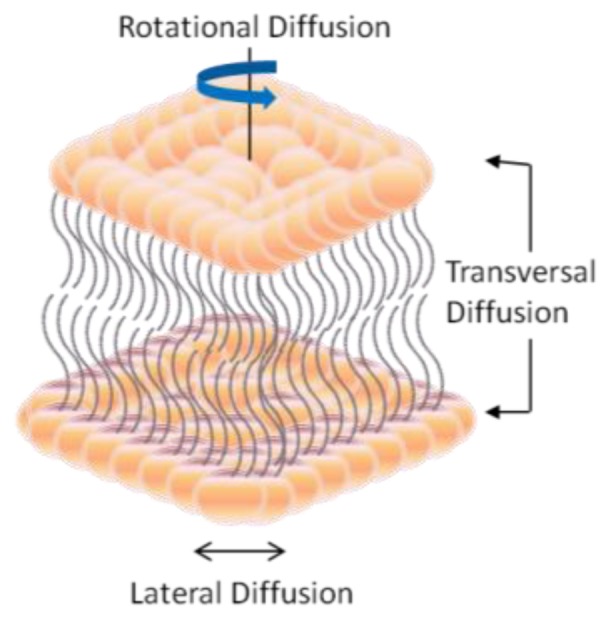
Diffusion mechanism inside the lipid bilayer membrane (LBM).

Biological membranes are dynamic structures [20,21]. It has been observed that both lateral and rotational orders of a lipid within the membrane bilayers are continuously changing with respect to time. Conformational changes affect the conformational order of a lipid molecule within the hydrocarbon chains. The lateral diffusion coefficient measures the ability of a lipid molecule to laterally exchange with one of its neighbors and rotational diffusion coefficient calculates the angular rotation of a lipid molecule around its axis perpendicular to the plane of the bilayer [21]. A special case of molecular dynamics is the transfer of one lipid molecule from one leaflet of the bilayer to the other one. Such cases demonstrate slow diffusion across the bilayer membrane, which is called transversal diffusion or flip-flop. It involves the rotation of the lipid molecule in the plane of the bilayer followed by its translation perpendicularly to the plane of the bilayer as shown in Figure 3. Muller and Herrmann reported [21] that proteins are limited to lateral diffusion within the bilayer membrane.

Methods have been developed for forming different types of lipid membranes include black lipid membrane (BLM), supported lipid bilayer membrane (s-LBM), air-stable lipid bilayer membrane (as-LBM), hybrid lipid bilayer membrane (h-LBM) and polymer-cushioned lipid bilayer membrane (pc-LBM). Each has advantages and disadvantages with regards to ease of formation, membrane lifetime, physiological limiting of fused transmembrane proteins. The following sections review each membrane type and its limitations.

### 2.1. Black Lipid Membrane (BLM)

Black lipid membranes were first described by Mueller *et al*. [22]. Lipid bilayer, a thicker annulus, and microlenes are the three components of the BLM. A thicker annulus usually forms at the interface between the supporting substrate and the bilayer [23]. A number of methods are reported to form BLM but the two most well-known methods include painting of the lipid solution over the aperture [21] and the formation of a folded bilayer [24].

**Figure 4:**
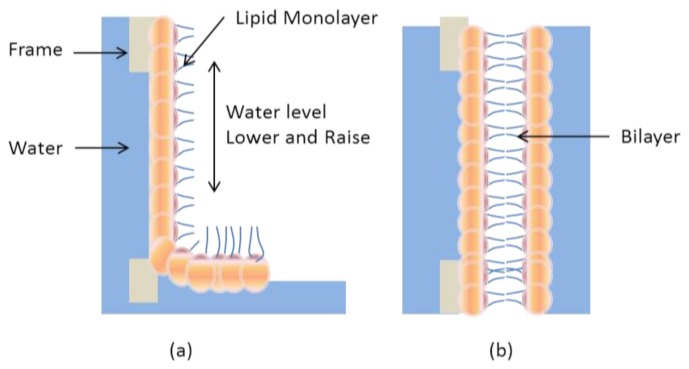
Black lipid membrane (BLM) deposition: (**a**) A single dip and pull of a mechanical support into water coated by a lipid results in a monolayer; (**b**) Lowering the substrate twice forms a BLM at the water interface.

To form suspended membrane, termed nano-black lipid membrane, the prerequisite is the deposition of the hydrophobic monolayer on top of the upper surface [25]. Since their advent, black lipid membranes have been used to investigate various biophysical processes. Peptides, proteins, antibiotics and other pore-forming biomolecules are used to form ion channels in phospholipid bilayers [26–28]. Figure 4 shows the formation of folded lipid membrane when the solution on the side containing a lipid monolayer is slowly lowered and then pulled up from the solution (Figure 4a). This procedure deposits a monolayer with each pass, thereby producing the black lipid film. Black lipid membranes (BLMs) patterned in this manner are suspended across the mechanical frame support. This provides a freestanding phospholipid bilayer into which transmembrane proteins may be fused. The resulting membrane is suspended directly on the surface liquid making the BLM the artificial membrane most similar to a living system. However, the surface tension required to form suspended bilayers over a fraction of a square millimeter is insufficient for long term stability of the membrane. As such, BLMs often have a usable lifetime of less than 1 h. Thus, short term definitive testing is the primary means by which information can be gathered from a biological test using BLMs. The most common methods used are electrical IV tests and simple light microscopy. For example, the thickness of black lipid membranes in aqueous solution can be deduced from measurements of reflected light intensity [29]. In addition, nonlinear optical microscopy, coupled with an appropriate dye molecule and correlated electrical measurements have provided quantifiable structure and dynamics of suspended lipid bilayers at the molecular level [30]. To improve the long term stability for measuring data and allow extensive evaluation, some supportive structure is required to sustain lipid bilayer membrane.

### 2.2. Supported Lipid Bilayer Membrane (s-LBM)

Solid substrates are often used to support the engineered phospholipid bilayers. These layers are more robust, stable, and open new opportunities for using surface specific analytical techniques, which were not available for BLM. Solid-supported lipid bilayers (s-SLBs) consist of a continuous lipid bilayer deposited onto a planar solid substrate. An ultrathin water layer separates the membrane from the solid surface by a distance of 10–20 Å [28]. In order to obtain high lipid mobility, the choice of substrate should be hydrophilic, smooth and clean. The best substrates used to form s-LBM are fused silica [31], borosilicate glass [32] and mica [33]. In addition, different methods have been used to deposit bilayer membrane on single crystals of TiO_2_ and Sr_2_TiO_2_ as well as on thin films of SiO_2_ on LiNbO_3_ crystals [34–36]. Skeletonized zirconium phosphonate modified surfaces can also be used to support planar lipid bilayers. The holes in the skeletonized film provide reservoirs beneath the bilayer providing opportunity for proteins to span the bilayer [37].

**Figure 5:**
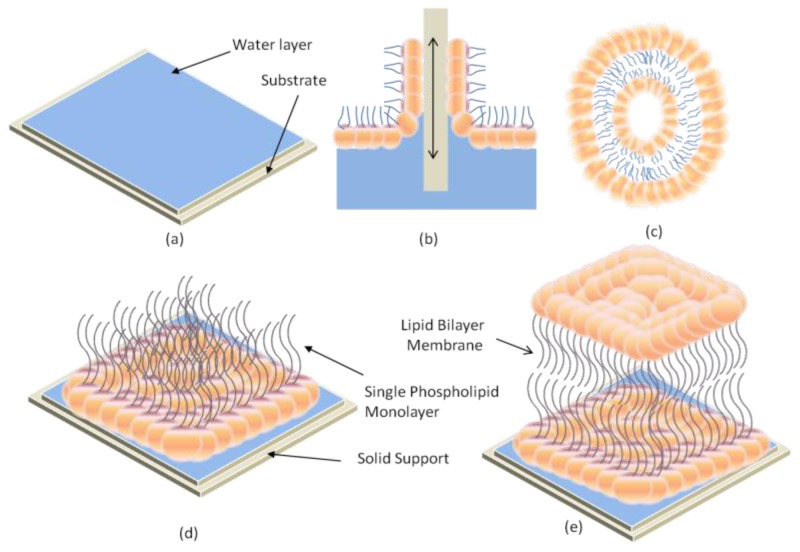
The formation process for lipid bilayer membrane on solid support surfaces using a combination of the Langmuir-Blodgett technique and vesicle fusion processes. (**a**) Substrate with water layer; (**b**) Pulling in and out hydrophilic substrate through a lipid monolayer; (**c**) Vesicle; (**d**) Substrate with single lipid monolayer; (**e**) Solid supported LBM.

There are three general methods for the formation of supported phospholipid bilayers on substrate. The first method involves transfer of amphiphilic molecules from the air-water interface to a solid substrate [38]. It involves the transfer of a lower leaflet of lipids from the air-water interface by the Langmuir-Blodgett technique. The transfer of an upper leaflet is attained using the Langmuir-Schaefer technique, in which the substrate is subsequently lowered horizontally onto the deposition medium. This method is useful for the formation of an asymmetric bilayer [39]. Incorporation of proteins onto the bilayer lipid with this technique is difficult because some portions of the proteins are exposed to air and can become denatured before transferring within the monolayers. The second method involves the adsorption and fusion of small unilaminar vesicles (SUVs) from an aqueous suspension to the substrate surface [40,41]. Different methods have been reported to prepare SUVs [42,43]. However, there are several factors which affect the adsorption and fusion of SUVs to solid supports. Most of them include the vesicle composition, size, surface charge, surface roughness, surface cleanliness, solution pH, ionic strength, and osmotic pressure of the vesicles [44,45]. Some researchers proved that the adsorption process can be accelerated by the presence of Ca^2+^ and Mg^2+^[37]. Fusion of SUVs to the substrate can also be enhanced by heating [46] and also by the addition of polyethylene glycol [47]. The third method is the combination of the first and second methods. Transferring of monolayer via Langumir-Blodgett technique is followed by vesicle fusion to form the upper layer [39]. A detailed description of this methodology is shown in Figure 5.

Recently, Tantawi *et al*. [48,49] demonstrated the same LB/LS technique to create the artificial phospholipid membranes. This technique offers the advantage of tightly packing the lipid molecules, hence resulting in a bilayer membrane that is as strong and tight as possible. The goal is to pack the lipids tight enough to form a contiguous layer that provides molecular ordering without supplying so much pressure that the lipids begin to fold over on themselves. Thus, one can directly control the surface pressure of the membrane desired for any specific biological test.

At low pH, lipid membrane spreading over a planar surface is favorable, regardless of the net charge on the bilayer [49]. The major advantage of using solid supports is to achieve high robustness and stability of the phospholipid bilayer membrane. However, the supported membrane is not truly decoupled from the underlying substrate, and solid supported phospholipid bilayers have some limitations in terms of their substrate compatibility. The interaction between the bilayer and supporting substrate can lead to shape changes and immobility of transmembrane or surface binding proteins which alter the physiological response of the system.

### 2.3. Air-Stable Lipid Bilayer Membrane (as-LBM)

**Figure 6:**
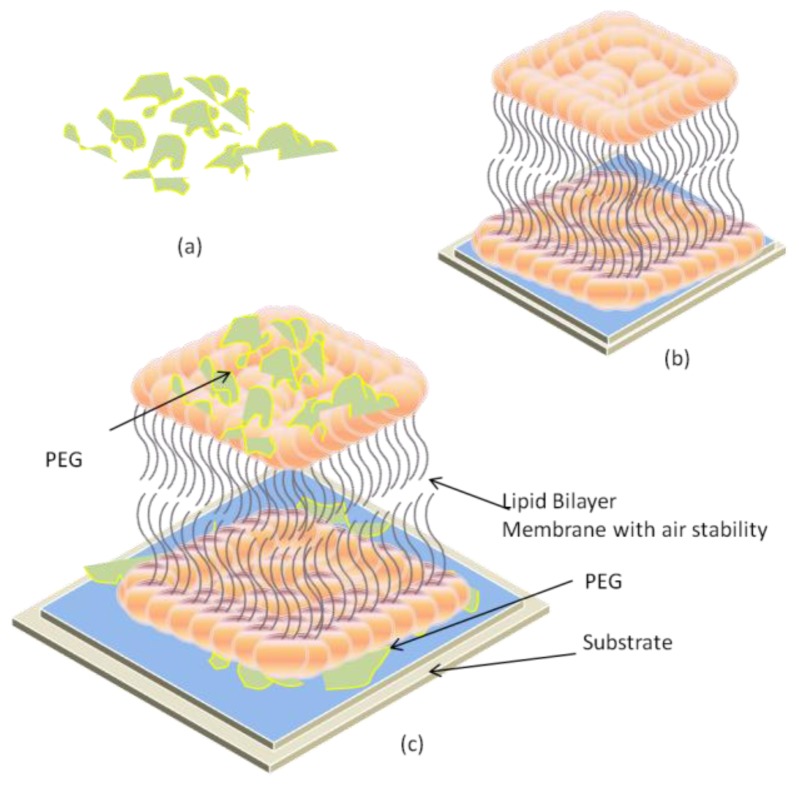
Air stability of a lipid membrane is increased using a Phosphatidylethanolamine (PEG) film. (**a**) PEG; (**b**) Simple solid supported LBM; (**c**) Vesicles with PEG on substrate.

Air-stable lipid membranes represent an emerging field in solid supported lipid bilayer membranes (LBM). Systems that support air stability include hybrid bilayers [50], protein stabilized lipid bilayers [51], and polymerized membranes formed using synthetic diacetylene-containing phospholipids [52,53]. However, these systems can suffer from either poor lipid mobility or are almost completely covered with protein. Both of these problems detract from the ability to employ the platform in sensing applications. Recently, an air-stable system was developed that maintains both high lipid mobility and is capable of binding analyte proteins to ligands by fusing vesicles containing polyethylene oxide oligomers conjugates of phosphatidylethanolamine (PEG) to substrate [54]. The presence of PEG in the membrane did not substantially hinder the binding of streptavidin to biotinylated lipids present in the bilayer. An illustration of the PEG stabilized membrane is presented in Figure 6.

The distance between the lower leaflet of the bilayer and the solid support is not sufficient to prevent lipid-solid surface interactions and ensuing frictions. This causes decreased in lipid mobility and denaturation of incorporated transmembrane proteins [55]. Normally, phospholipid bilayers shrink and crack upon cooling [56], which is potentially a problem for making sensor devices from supported membranes under certain conditions; however, this issue was overcome by Zhang *et al*. [57]. They successfully showed that when positively charged lipids were added to dimyristoylphosphatidylcholine vesicles, phospholipid bilayers on mica surfaces would resist cracking upon cooling into the gel phase. This promotes storage for longer periods of time than otherwise possible with supported lipid bilayers.

**Figure 7:**
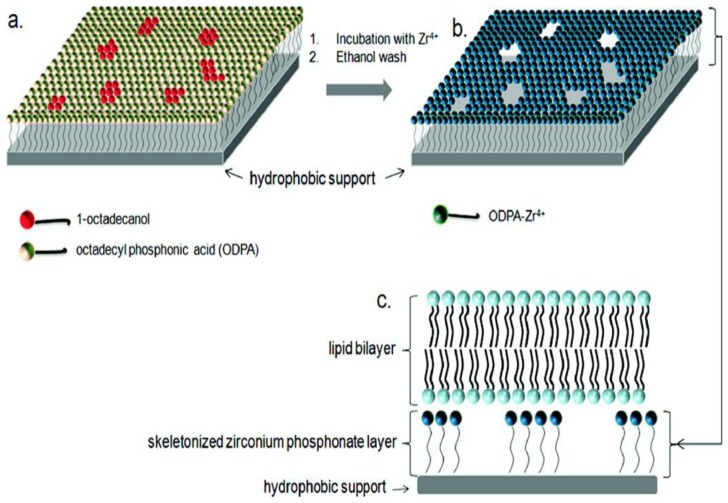
Supporting lipid bilayers with skeletonized zirconium phosphate surface (**a**) A mixed monolayer is on a hydrophobic surface; (**b**) the surface is washed with ethanol to remove the non-covalently bound molecules; (**c**) Lipid bilayers are formed on the skeletonized surfaces by vesicle fusion or by Langmuir-Blodgett/Langmuir-Schaefer techniques. Reprinted with permission from Langmuir [37]. (American Chemical Society, 2012).

A method which describes the forming of fluidic and air-stable LBM through tethered and dispersed cholesterol groups incorporated into the bottom leaflet was reported by Deng *et al.* [58]. Achieving air stability allows us to easily fabricate SLB microarrays from direct robotic spotting of vesicle solutions. A facile method for the formation of air-stable phospholipid membranes on silicon dioxide surface was first reported by Zhang *et al*. [59]. The process was further improved through the use of skeletonized zirconium phosphonate surfaces by Fabre *et al.* [37] to support planar lipid bilayers. In this case, the surface mobility of the interlaminar phosphonate layers provided reservoirs beneath the membrane to incorporate transmembrane proteins allowing the BK channel protein to insert directly into a preformed bilayer on the skeletonized surface. To achieve the reservoirs, skeletonized zirconium phosphonate films are prepared using the Langmuir-Blodgett (LB) technique as shown in Figure 7.

### 2.4. Hybrid Lipid Bilayer Membrane (h-LBM)

**Figure 8:**
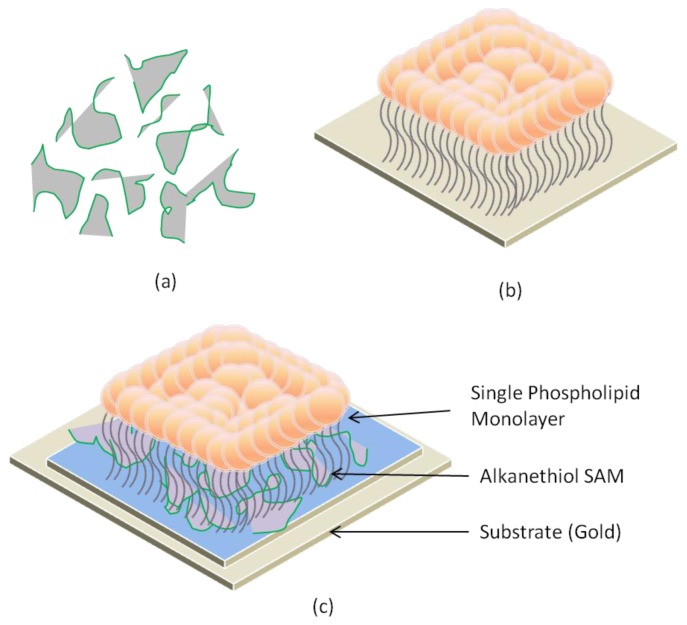
Illustration of a hybrid lipid membrane. (**a**) Alkanethiol SAM layer; (**b**) A single phospholipid monolayer on substrate; (**c**) Alkanethiol SAM layers arrange themselves over the gold surface, providing single layer lipid adhesion.

Hybrid phospholipid membranes are more robust than the solid supports because they have strong interactions between the alkanethiol self-assembled monolayer (SAM) layer and the substrate as shown in Figure 8. They can be dried and rehydrated when formed at an air–water interface by keeping some of the properties unchanged [60]. The problem with this approach was that the alkanethiol SAM layer is more crystalline in structure when compared to a normal leaflet of a phospholipid bilayer, which results in less fluidity of the membrane and restricts the ability of proteins to fuse randomly. Hydrophobic SAMs support the formation of hybrid bilayers in which a lipid monolayer was adsorbed at the interface [61]. Furthermore, protein incorporated into the bilayer is also incompatible with the Au support layer [62]. This can restrain its diffuse migration of lipids in the bilayer. It has been concluded that it is difficult for hybrid lipid bilayer membranes to accommodate the transmembrane proteins with both large extracellular and intracellular domains. Tethered bilayer lipid membranes (tBLMs) (exhibit higher lipid diffusion coefficients relative to SAMs with saturated alkyl chains) have been used extensively to investigate membrane proteins and processes such as redox activity [63], ion transport across the membrane [64], antibiotic binding [65], and other applications [66–68].

### 2.5. Polymer-Cushioned Lipid Bilayer Membrane (pc-LBM)

**Figure 9:**
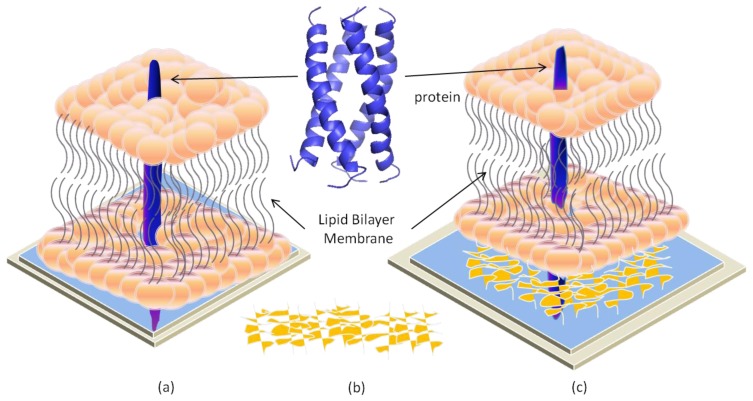
Incorporation of a protein in lipid bilayer membrane with polymer cushion on solid substrate. (**a**) Denaturation of transmembrane protein when comes in contact with substrate; (**b**) A polymer cushion; (**c**) Polymer cushion protects the protein from the substrate surface.

Black lipid membranes, solid supported lipid bilayers and SAM/monolayers have been used to mimic many cellular processes; however, it remains difficult to engineer the appropriate environment for transmembrane proteins. In artificial membranes, the water layer between substrate and bilayer does not prevent the protein from interacting with substrate, which results in denaturation of proteins. Therefore, the incorporation of transmembrane proteins into supported lipid bilayers has not yet been satisfactorily explored. This is largely due to the limited space between the bottom leaflet of the bilayer and the solid supporting substrate. Typically, the distance between the bilayer and the underlying solid substrate is only around 1 nm. Due to this limited space availability, proteins may stay inside the bilayers, leading to substantial damage of lipid bilayer membrane (LBM) as shown in Figure 9a. In the case of a thin water layer, the separation distance between the substrate and the biomembrane is typically 10–20 Å [28]. This presents a problem for transmembrane proteins, which can become immobilized by direct interaction with the solid support [69]. The different strategies for separating a LBM from a solid surface were reviewed by [70–72]. These studies concluded that soft hydrophilic polymer cushions could be introduced between the hard inorganic substrate and the LBM to completely avoid the possible substrate-lipid interactions and may affect the protein structure or function [73]. Therefore, researchers found an alternative technique to support the LBM using polymers (called polymer supported bilayer systems) as shown in Figure 9c. These experiments proved that a polymer layer can keep the membrane away from substrate. Polymer support must be hydrophilic, soft and not highly charged.

In the last two decades, several types of polymer cushions have been explored as supporting templates for bilayer lipid membranes. One example is the use of a thin hydrogel as the polymer layer on indium–tin oxide (ITO), with mesh sizes smaller than the size of the protein used to form the protein-tethered bilayer lipid membrane. The thickness of these polymer cushions was no more than 100 nm [56]. Two important polymers cushions used are lipopolymer and polyelectrolyte [74–76]. However, in some experiments, polydopamine, dextran, cellulose and chitosan [77–80] were also reported as cushions.

**Figure 10:**
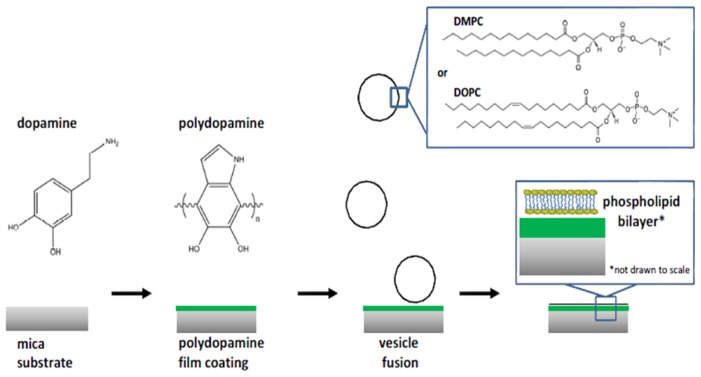
Preparation of polydopamine supported membranes and the structure of polydopamine. Reprinted with permission from Science [79] and Journal of Physical Chemistry C [82].

Several techniques are reported to form the bilayer on polymer cushion. One of them is vesicle fusion or Langmuir-Blodgett/Langmuir-Schaffer transfer. These systems allow protein incorporation in non-denaturing conditions. However, it has also been reported that the large number of tethering molecules can decrease the mobility of the number of supported phospholipid bilayer, which further changes the transition temperature. Some scientists also prefer oxide substrates to polymer supported phospholipid membranes because they are more stable and possess fewer surface defects [81]. A poly(l-lactide acid) (PLLA) thin film was introduced as a soft polymeric cushion between the CaF_2_ substrate and the lipid bilayer assembly. To validate this approach, sum frequency generation (SFG) vibrational spectroscopy was used to examine and compare single lipid bilayers assembled on the CaF_2_ prism surface and on poly(Lactic acid) (PLLA) cushion [82]. They demonstrated that hydrophilic PLLA cushions are indeed promising substrates to support single lipid bilayers. Other soft polymer cushions such as polydopamine have also been reported. Nirasay *et al*. [83] deposited zwitterionic phospholipid bilayer on the polydopamine cushion by fusion of dimyristoylphosphatidylcholine (DMPC) and dioleoylphosphatidylcholine (DOPC) vesicles as shown in Figure 10. They prepared polymer-supported membranes over a continuous polydopamine film (20 nm) and assessed their fluidity.

It is important to note that lipid membranes typically require different surface pressures to meet the physiological requirements for proteins. Thus, the author proposes that the use of the Langmuir-Blodgett (LB) technique and LB/LS techniques will provide more control over bilayer deposition that is currently demonstrated in the majority of research presented to date [48,49]. This technique offers the advantage of tightly packing the lipid molecules, hence resulting in a bilayer membrane that is as strong and tight as possible. One concern is that LB films are artificially created and not derived from living systems like cell-spliced membranes. However, control over the surface pressure may pose a significant benefit to biological research on the packing density of various proteins within lipid membranes and rafts.

## 3. Probing Transmembrane Proteins on Solid Surfaces

Membrane proteins play a major role in every living cell. Protein adsorption on solid surfaces has long been an attractive subject for research in the field of natural sciences, biosensors and protein chips, medical implants and in the food industry [83,84]. Both the protein and the surface properties influence protein–surface interactions. Important parameters include surface energy, polarity, charge, and morphology [85]. Membranes have been used to investigate protein adsorption phenomena with a realistic background from biological systems [86]. Atomic force microscopy, scanning electron microscopy, confocal microscopy and other surface topography equipment can be used to measure the surface tension, polarity, charge and many other parameters [87–90]. It has been observed that proteins tend to adhere more strongly to nonpolar than to polar molecules, to high surface tension than to low surface tension and to uncharged than to charged substrates. The affinity of proteins to surfaces increases on hydrophobic substrates and decreases on hydrophilic substrates [91]. For instance, glycoproteins adsorb extensively on hydrophilic planar surfaces and sparsely on hydrophobic surfaces [92]. Cha *et al*. [93] reported that uncharged vesicles made from zwitterionic lipids appear to fuse more rapidly to substrates at basic pH and normal ionic strength as compared to negatively charged vesicles. Investigation of fusion and spreading of phospholipid bilayers on glass surfaces is done as a function of pH and ionic strength.

**Figure 11:**
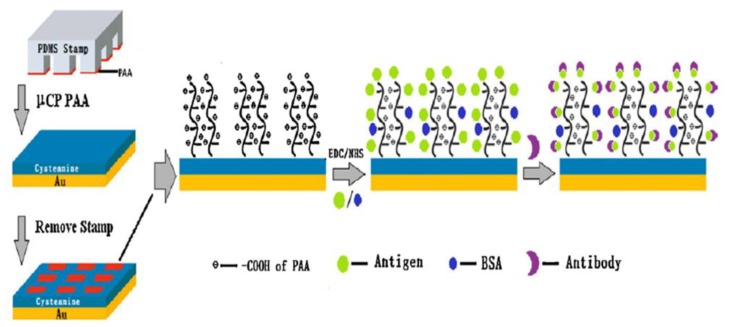
Schematic diagram of patterning Poly(acrylic acid) (PAA) brushes and immunoassay along SPRi process. Reprinted with permission from Applied Surface [95].

In recent years, the favorable protein adsorption properties of Poly(acrylic acid) (PAA) brushes have been reported in different publications [92,94,95]. At low salt concentration, these polyelectrolyte layers retain proteins in their native state. In addition, the activity of the enzyme horseradish peroxidase was not affected after adsorption to PAA brushes in contrast to adsorption to a bare silica surface [96]. Moreover, the protein insulin does not transform into amyloid fibers when attached to PAA brushes even at conditions at which fibrillization is normally observed [97,98]. In contrast to low salt concentrations, PAA brushes become highly protein resistant at high salt concentrations as these conditions suppress the process termed “counterion evaporation” which is the major driving force for protein adsorption to polyelectrolytes [99]. Recently, a facile micro contact printing (uCP) method for creating the pattern of 3D PAA polymer brushes on gold surface is demonstrated by Wang *et al*. [95], in which model protein (H-IgG) and disease related protein (HBsAg) are grafted, as shown in Figure 11. The antibody/antigen interactions on these polymer brush patterned surfaces were monitored by fluorescence microscopy and surface plasmon resonance imaging (SPRi). The chip shows highly specific and tremendous signal amplification compared to the PAA brush surface without patterning. Moreover, this surface can effectively prevent non-specific adsorption of proteins.

### 3.1. Parameters Affecting Protein Interaction with Substrate

Parameters which highly affect the proteins during interaction with solid substrate are: temperature, pH, ionic strength, adsorption behavior, conformational reorientation, buffer composition and surface density. If true physiological conditions were to be mimicked, these parameters could easily be fixed but many experimental studies were conducted at arbitrary conditions.

Temperature and pH affect the protein adsorption. At high temperature, increased adsorption rates can be expected due to the accelerated diffusivity of proteins towards the sorbent surface [100]. The pH determines the electrostatic state of proteins. When pH equals the isoelectric point (pI) of a protein, the numbers of negative and positive charges are in balance resulting in a net neutral molecule [101]. At low pH conditions, proteins are positively charged and are negatively charged at high pH conditions. Efficient screening of the protein’s electric potential reduces lateral interactions. This in turn may initiate an increase in packing density, suspension (dispersion of molecules in medium but they are not dissolved) of cooperative effects, and protein–protein repulsions [102]. Moreover, high ionic strength conditions increase the tendency of proteins to aggregate [103].

Conformational reorientations of proteins within bilayer surfaces were investigated by Norde [104,105]. They noted that small, rigid proteins have a low chance for structural realignment upon surface adsorption. On the other hand, intermediate size proteins are usually able to undergo conformational reorientations when interacting with a lipid surface. It is now generally accepted that many proteins undergo conformational changes upon adsorption to a solid interface. These changes are due to the fact that the conformation of a protein that corresponds to the free energy minimum in solution typically does not correspond to the free energy minimum of this protein once it is in contact with the surface. The conformational and/or orientational changes that occur upon adsorption are expected to affect the biological function of the protein. Lipoproteins are structurally labile and therefore show a strong affinity to hydrophobic surfaces with significant conformational reorientations. On the contrary, adsorption of glycoproteins on hydrophobic surfaces is hindered due to the high content of hydrophilic glycans [106,107].

The final layer thickness of a protein monolayer in its saturation state is higher in the case of “end-on” oriented proteins than in the case of “side-on” oriented proteins which were investigated by Lu *et al*. [108] and Su *et al*. [109]. After extensive simplification, the protein is represented as globular entity consisting of positive and negative domains. At low surface densities, the protein orientation is solely determined by surface–protein interactions while at high surface densities, increasing protein–protein interactions can trigger orientational changes leading to a decrease of protein–surface interactions [110–112].

### 3.2. Adsorption Mechanisms

Adsorption behavior is usually the result of an overlapping of transportation, adsorption and repulsion processes. It has been observed that small proteins diffuse quickly as compared to large ones. Therefore, these small proteins are major species in the early adsorption stage. Because of the large contact area, larger proteins bind stronger to the surface and can easily repel other pre-adsorbed proteins. As a consequence, the total mass of adsorbed proteins can pass through a local maximum during the course of adsorption [113]. Adsorbed proteins change their orientation from one state to another by reducing attractive forces towards the surface and repulsive forces between neighboring proteins. The orientation of a structurally stable protein on a surface is characterized by “side-on” or “end-on” orientation, referring to an elliptically shaped particle that is respectively attached with its long or short axis to the surface [114]. Adsorption can also stabilize the structure of some proteins and hence improve their resistance to denaturation as compared to dissolved proteins [115]. Also, it was shown experimentally that the activity of enzymes after surface adsorption can be indifferent [116] or reduced [117] compared to dissolve enzymes if the orientation of the active sites is directed toward the solution or toward the surface, respectively. Some proteins or peptides exhibit their function only after adsorption [118]. By contrast, adsorption can also lead to the irreversible alteration of proteins that do not refold into their native structure after desorption.

Proteins are typically asymmetric complex molecules of a few nanometers in size. Rabe *et al*. [86] observed that proteins rotate freely in solution and on lipid surfaces. Each protein change is conformation to align nonpolar regions within the lipid bilayer and polar regions on the membrane surface and bulk solution. A protein’s surface can be divided into different patches and each patch can be of hydrophobic, hydrophilic, positively, or negatively charged nature [119,120]. Therefore, proteins predominantly expose those patches that are rich of hydrophilic residues towards the hydrophilic surface and the opposite happens on hydrophobic surfaces. Similarly, proteins adsorbing at positively or negatively charged interfaces tend to expose oppositely charged regions to the surface [121,122]. It has been observed that proteins not only interact with the sorbent surface but also with one another in lateral direction. This occurs when proteins of the same species orient such that a net charge of equal sign causes long range inter protein repulsions [123]. Thus, one can theoretically conclude that larger proteins tend to repel smaller ones from the sorbent surface [101] In addition, there are some proteins which diffuse in close proximity to the surface and they will more likely to adsorb if there are already pre-adsorbed proteins.

**Figure 12:**
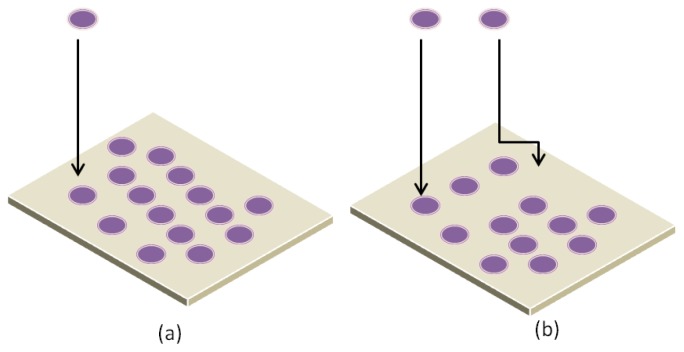
Non-cooperative and Cooperative adsorption mechanisms. (**a**) Non-cooperative adsorption: proteins track vertically towards the surface; (**b**) Cooperative adsorption: proteins track vertically and horizontally towards the surface.

It was Chatelier *et al*. and Minton *et al*. [124–126] who provided the first complete definition of the terms positive cooperative adsorption, negative cooperative adsorption, and apparent non-cooperative protein adsorption. During cooperative adsorption, proteins are vertically tracked toward the surface and at the same time horizontally repelled by neighboring proteins as shown in Figure 12.

**Figure 13:**
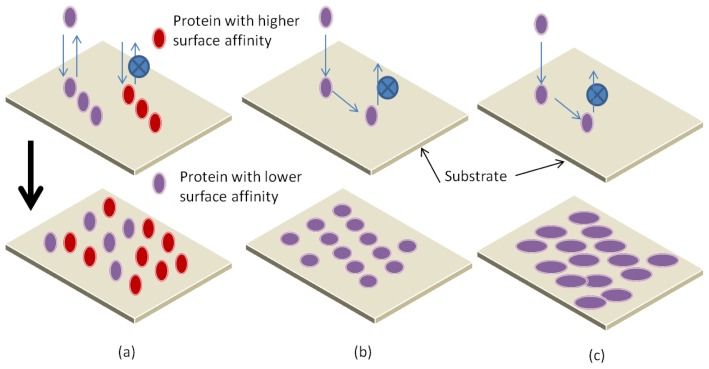
Adsorption Kinetics of proteins with surface. (**a**) Proteins with higher surface affinity replace the proteins with lower surface affinity; (**b**) Change of the protein’s orientation after adsorption on surface; (**c**) Proteins with end-on orientation allows more species to adsorb on the surface.

At equilibrium stage, the number of adsorbing proteins is equal to the number of desorbing proteins. In the case of pure irreversible adsorption, proteins cannot be adsorbed to the surface anymore as all binding sites are occupied. A rather unexpected phenomenon is the observation of an overshoot during the adsorption kinetics. This overshoot refers to a situation where the adsorption kinetics passes a local or global maximum before the saturation is reached. Different techniques are used to observe overshooting protein adsorption kinetics [116,118,127]. Three different explanations for overshooting adsorption kinetics are shown in Figure 13. First, The Vroman effect describes the adsorption of a fast adsorbing species with low surface affinity and a slowly adsorbing species with high surface affinity [127,128]. They concluded that proteins with higher surface affinity replace the proteins with lower surface affinity. The second was given by Daly *et al*. [112], which is based on a change of the protein’s orientation after adsorption on surface (hydrophilic). Finally, Wertz and Santore [110] showed that the initial end-on orientation allows more species to adsorb on the surface (hydrophobic) as the final and energetically preferred side-on orientation.

A highly important aspect connected with protein adsorption is the aggregation of proteins into oligomers of a few monomers or into clusters of up to several hundreds of protein monomers. This process accommodates protein adsorption at solid interfaces and promotes the adsorption kinetics. Aggregation or clustering can be vital for the control of signal transduction pathways [118,129,130] or show signs of complex cellular functions [131]. The application of Forster Resonance Energy Transfer (FRET) spectroscopy has revealed that protein clusters can deposit from the solution onto the surface and it starts spreading. The spreading rate completely depends on the type of surface.

For fast spreading, hydrophobic surface is used with high surface mobility, and for slow spreading, hydrophilic surface is used with low surface mobility [132]. A detailed model describing the likely dependences of the rate constants of protein adsorption and desorption on the fraction of charged lipids in a lipid bilayer are described in Trusova and Gorbenko [133], Rabe *et al*. [134] and Zhdanov and Kasemo [135].

Tethered bilayer lipid membranes have been used as a model system to mimic the interactions between the whey protein β-lactoglobulin and a lipid interface. Lipid composition as well as the structural properties of the protein governed their interactions, which were probed by a combination of surface plasmon spectroscopy, neutron reflectivity, and electrochemical impedance spectroscopy. A comparison of results obtained using native and a partially unfolded protein indicate that the protein preferentially forms loosely packed layers at the lipid interface [136].

Landau theory was used to describe the decay of order in a lipid bilayer as a function of distance from the embedded protein and to consider only the substantial fraction area of protein that is incorporated with the membrane’s surface area [137,138]. Jihnig *et al*. later extended this theory to large protein concentrations [139]. The thermodynamic properties of several different types of lipid-protein interactions were later calculated by Scott JR and Coe [140]. They described the orientation change in terms of phase transition inside the lipid-protein interaction with respect to temperature and fluidity.

The spontaneous reconstitution of lipid-protein complex was examined by Scotto and Zakim [141]. Spontaneous insertion of proteins into dimyristoylphosphatidylcholine vesicles was facilitated by sonication at 4 °C. Fusion between protein-free and protein-containing vesicles in a liquid crystalline phase was extremely slow. It shows that the spontaneous insertion of pure membrane proteins into preformed vesicles can be a superficial event. Moreover, the incorporation of a pure membrane protein into a preformed lipid bilayer did not occur during fusion between vesicles. Solid-supported lipid layers are able to incorporate membrane proteins only if an aqueous layer will separate the lipid layer from the substrate [142]. Furthermore, the mechanical role of the lipid bilayer in ion channel conformation and function with specific reference to the case of the mechanosensitive channel of large conductance was also discussed in detail [143,144].

## 4. Transmembrane Protein Fused in Bilayer Lipid Membrane on Porous Silicon (PSi)

Multiple groups have recently investigated transmembrane proteins fused into lipids supported by a variety of substrates. Gold [145–148], alumina [26,149,150], Teflon [151,152], porous silicon (PSi) [153–162] and many others materials have been successfully used as supporting surfaces (substrates) to study proteins. Each substrate has its own advantages and disadvantages when used as support for lipid bilayer membranes (LBMs). Because these materials affect both the lipid and fused protein, they may have similar but not identical properties to the *in vivo* one.

Researchers have been working to provide the best solution for the specified problem and monitor the proteins under native environment as compared to non-native. The life time of LBM is reduced with Teflon as supporting material and at high temperature; it emits toxic particles and gases. Porous alumina has poor biocompatibility. Also, the high aspect ratio of porous alumina is a disadvantage. Using gold as coated material, capacitance is reduced which further decreases the duration of the experiment. Mica as substrate gives low conductivity and it is impossible to record the real time monitoring of lipid-protein interaction and ion channel monitoring. Also, to record the image using atomic force microscopy (AFM), and if the surface is mica, the interaction force between the cantilever and the biological object will decrease. One of the best possible solutions is to use porous silicon, PSi, to keep the transmembrane proteins in an environment as close as possible to the *in vivo* one. In addition, PSi has been highly used in drug delivery and other biological labels due it its high surface-area-to-volume ratios [161–169]. Moreover, its compatibility with electronic devices makes it more attractive for medical and electronic applications over other materials. Other properties include its low cost, electroluminescence, and changes in refractive index by varying the depth and size of pores [170]. It has high biocompatibility, biodegradability and photoluminescence; which allow it to be used for an *in vivo* monitored drug delivery [171–175].

Pore size of porous silicon can be precisely controlled by fabrication parameters. The mechanism for the formation of lipid bilayer membrane is dependent on the pore size, because small pores are required to achieve the lower surface coverage of adsorbed lipid vesicles. Therefore, pore size dependent observations are suggested to be due to the hydrophilicity of the surface, which decreases with increases in the pore size [161]. To fabricate pores (holes), different methods have been documented. One of the most common methods was proposed by Goncalves *et al*. [153]. They proposed a silicon bed with electron-beam patterned holes in it to support native membrane layers of *C. Glutamicum*, but to fill the holes they had no access to the solution. Another method was given by Thibault *et al*. [157]. They used electron-beam lithography and reactive ion etching to develop holes, which are expensive and time consuming processes. Fabricated pores were interconnected inside the silicon bed and used side openings for access to the water solution. However, their methodology does not allow patterning of biological materials on the backside of the membrane.

Fertig *et al*. used planar microstructured through-water glass vials with pore diameters of 1–2 μm to detect the activity of single ion channel protein. Planar patch clamp electrodes were used to attain an electrical resistance higher than 1 G*Ω* between the membrane and the pore. The accessibility of the ion channel containing membrane can be enhanced by their experiments [158]. For protein immobilization, Simion *et al*. [156] used the electro-chemically fabricated porous silicon as a substrate. Worsfold *et al*. [154] also used porous silicon to support lipid bilayers for biosensing. However, these pores were not etched through the substrate and thus were closed from underneath, and this prevented the testing of transmembrane ion channels. We conclude that silicon membranes should be porous all the way through to be used as a support for biological membranes on one or both sides of the silicon structure.

**Figure 14:**
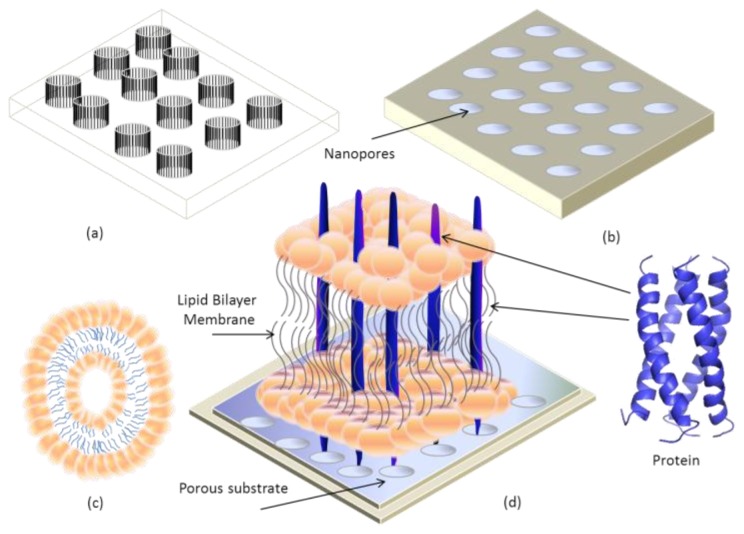
Incorporation of transmembrane proteins fused into lipid bilayer on porous structure. (**a**) Geometry of porous structure; (**b**) Prototype of fabricated porous structure; (**c**) Vesicle; (**d**) Lipid bilayer membrane on PSi.

**Figure 15:**
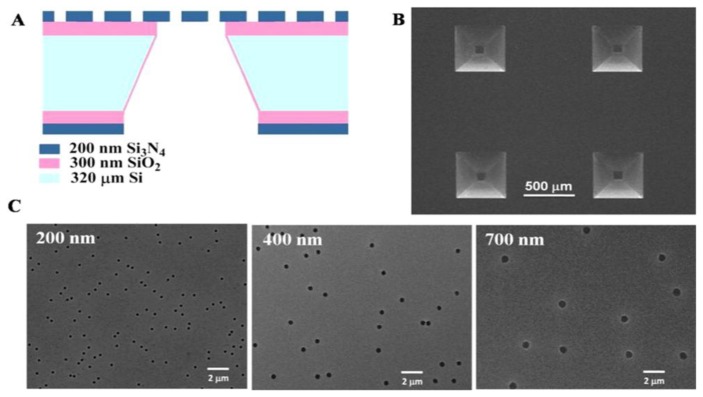
(**A**) Suspended nanoporous Si_3_N_4_ membranes; (**B**) Bottom-view of Si_3_N_4_ membrane using SEM; (**C**) Top-view of nanopores of Si_3_N_4_ membranes with different diameters using SEM. Reprinted with permission from Langmuir [155].

Currently, research groups have begun working to incorporate transmembrane proteins with lipid bilayer membrane on silicon devices, which must be porous throughout. A schematic illustration of this concept is presented in Figure 14. Recently in 2012, Zhu *et al*. [155] fabricated different nitride pores of size 200, 400 and 700 nm in diameter on one side of the Si wafer as shown in Figure 15. They used 4 inch n-type Si (100) wafers of 320 μm thick with both sides grown successively with 300 nm thick silicon oxide (SiO_2_) and 200 nm thick silicon nitride (Si_3_N_4_) layers. On one side of the Si wafer, negatively charged polystyrene, PS, colloidal particles of 200, 400, and 800 nm in diameter, respectively, were introduced to the surface of Si_3_N_4_ layer via electrostatic self-assembly, which was followed by deposition of a thin Cr mask layer. After stripping off the PS particles, inductively coupled plasma (ICP) etching was applied to remove the Si_3_N_4_ layer of the exposed regions. On the other side of the wafer, bulk Si and SiO_2_ layer were etched to create four trapezia shaped cavities by standard Si fabrication procedures including photolithography, Cr etching, anisotropic Si wet etching, and BOE (buffered hydrofluoric acid) wet etching, resulting in randomly distributed nanopores on the silicon nitride layers [155].

**Figure 16:**
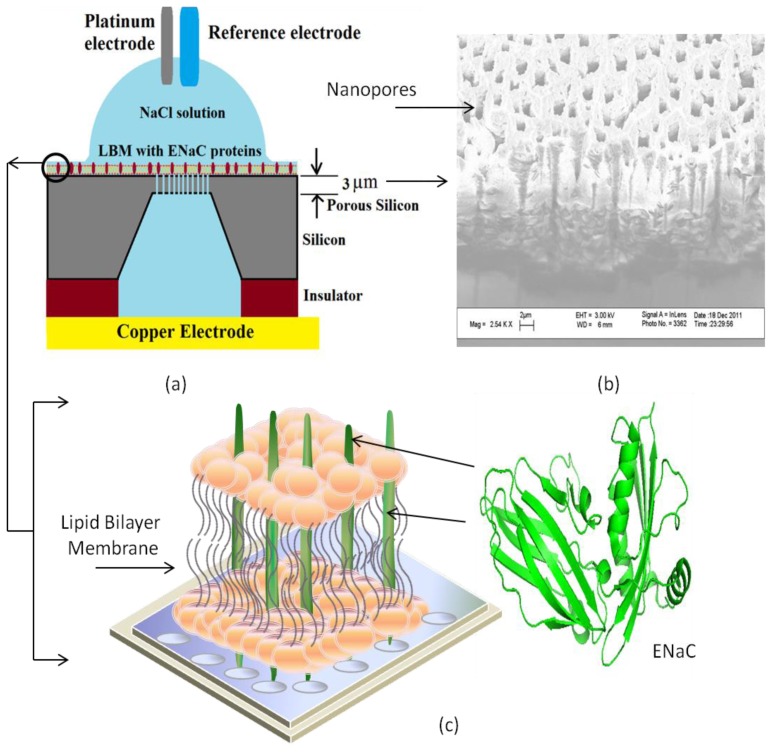
Illustration of experiment for porous silicon (PSi) device used for investigation of transmembrane protein fused in LBM. (**a**) Electrochemical cell formation; (**b**) Top-view of fabricated porous silicon using SEM; (**c**) Insight view of incorporation of ENaC with LBM on PSi.

Alternatively, Tantawi *et al*. [48,49,159] have chosen to pattern the porous silicon membrane using silicon on insulator (SOI) substrates. They successfully fabricated pores in the range of 0.2–2.0 μm using an electrical fabrication technique, and tested the supported bilayer with and without fused transmembrane proteins. A summary is presented in Figure 16. They used Langumir-Blodgett and Langumir-Schaefer techniques to prepare artificial lipid bilayer membrane on porous silicon structure. The Epithelial Sodium Channel (ENaC) transmembrane protein is then fused into it by direct spreading into the lipid bilayer. This work offers new capabilities as compared to most of the other silicon-based technologies [153,154,157]. This device can be used as a barrier between two different and fully accessible compartments, which provides access to the solution on both sides of the porous silicon membrane for the study of functional ion channels. We assert that porous silicon supported artificial membranes may also allow biologists to study protein-protein interactions in an environment as close as possible to an *in vivo* one.

## 5. Electrochemical Impedance Spectroscopy

Electrochemical Impedance Spectroscopy (EIS) has been used to characterize porous substrate platforms with deposited lipid bilayers and incorporated transmembrane proteins. This methodology is based on complex impedance measurements at a wide range of frequencies. Important parameters to analyze using EIS are the capacitance and resistance of the membrane, fused protein, and this technique has also been used to monitor the bilayer incorporation of a ligand gated ion channel protein and the modulation of its channel activity by the selective binding of an antibody [65,176]. For these tests, a periodic voltage signal of variable frequency is applied to the electrodes. The absolute value of the current response of the system is analyzed in terms of the complex impedance and the phase shift. Capacitance of the lipid bilayer membrane (LBM) increases when an electric field is applied. Hydrophobic pores form on the LBM, but do not contribute in the conductance mechanism. After the removal of electric field, LBM returns to its low conducting state, and we call this phenomena “reversible electric breakdown” [176]. Electric breakdown of the membranes can be measured using the voltage-clamp technique, which was presented by Chernomordik *et al*. [177]. Presence of cholesterol decreases the membrane fluidity and causes it to be more rigid [178]. One should expect that the incorporation of protein channels into the lipid bilayer membranes, pore formation in the membranes is stabilized but may increase at the vicinity of the proteins [176,178].

**Figure 17:**
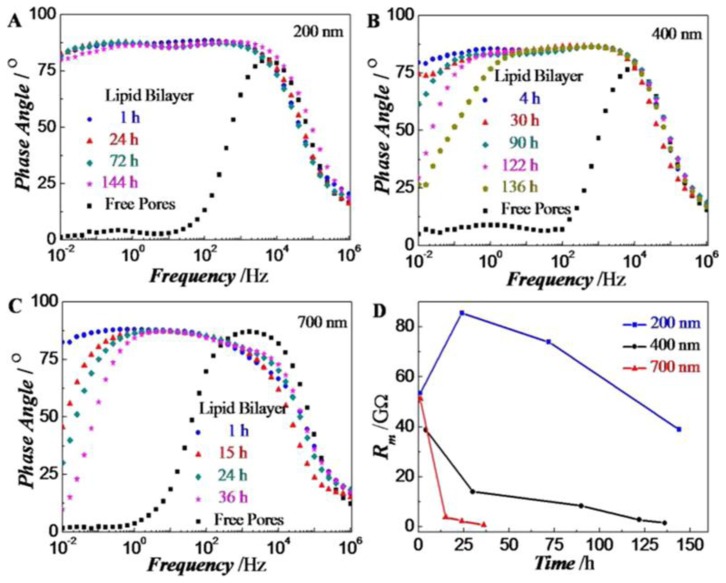
Electrochemical impedance spectroscopy (EI)S of pore-spanning nano-BLMs on silicon substrate with diameter (**A**) 200 nm; (**B**) 400 nm; (**C**) 700 nm at different time periods; (**D**) Membrane resistance at different time periods with different nano pores in a frequency range of 10^−2^–10^6^ Hz. Reprinted with permission from Langmuir [155].

Zhu *et al*. [155], Lundgren *et al*. [179] and Tantawi *et al*. [48,159] have all applied EIS to investigate the physical behavior of pore spanning lipid bilayer membrane on substrate’s surface with respect to time. Zhu *et al*. reported the mechanical stability and the lifetime of pore spanning nano black lipid membrane (BLM). They investigated electrical properties of nano-BLMs in terms of membrane resistance and membrane capacitance using electrical impedance spectroscopy (EIS) approach. They measured EIS at bias of 0, 0.05 and 0.10 V in a frequency range of 10^−2^–10^6^ Hz with pore diameters of 200, 400 and 700 nm. Zhu *et al*. recorded membrane resistance over time and concluded that with a 700 nm pore size, they had a resistance of 51.3 GΩ after 1 h of pore spanning nano-BLMs on porous silicon device. However, this high value was dramatically decreased to 2.4 GΩ and 0.8 GΩ after 24 h and 36 h, respectively, as shown in Figure 17C. For pores with a size of 400 nm, maximum resistance recorded after 122 h was 8 GΩ as shown in Figure 17B. As the pore was decreased to 200 nm, the membrane’s resistance increased to 53.6 GΩ after 144 h. These experimental results revealed an interesting finding that the life time of black lipid membrane can be improved by reducing the pore size. Figure 17D shows the detailed results at different time instances. They measured 38.8–53.6 GΩ BLM resistance and average specific capacitance of 5.9 μFcm^−2^ depending on the pore size. The life time of pore spanning nano-BLMs with pore sizes 200 and 400 nm were much higher as calculated by Drexler and Steinem [180] and Romer and Steinem [25] with same lipid 1,2-dioleoyl-sn-glycero-3-phosphocholine (DOPC) and alumina as substrate. This further supports the use of suspended BLMs over very small diameter pores.

**Figure 18:**
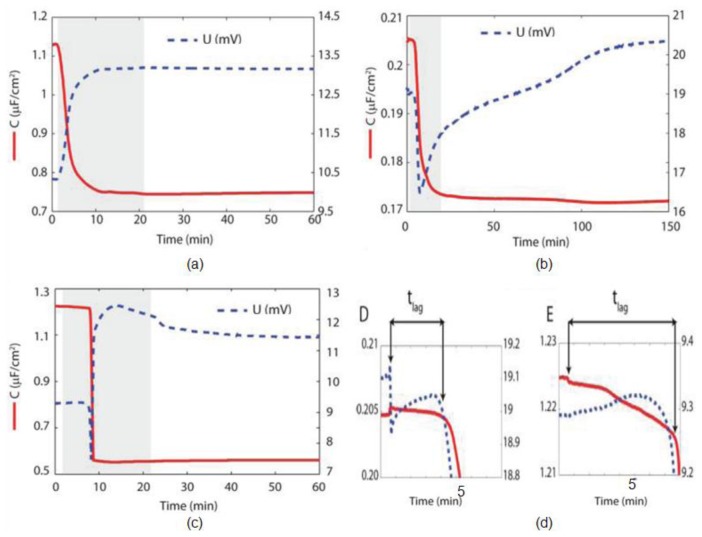
Change in capacitance and peak voltage (amplitude) upon insertion of vesicles on substrate. (**a**) POEPC vesicles on ODT SAM; (**b**) POEPC vesicles on 30 nm SiO_2_; (**c**) POPC vesicles on 5 nm SiO_2_; (**d**) Behavior change in capacitance and amplitude of case; (**b**) and (**c**) in first 8 min after introduction of vesicles. Reprinted with permission from Langmuir [179].

Lundgren *et al*. [179] measured EIS data of the other lipid bilayer and studied capacitance changes upon insertion of vesicles in the membrane with respect to time. They demonstrated real-time recordings in the layer formation process of bilayer made from (i) positively charged lipid 1-palmitoyl-2-oleoyl-sn-glycero-3-ethylphosphocholine (POEPC); (ii) neutral lipid 1-palmitoyl-2-oleoyl-sn-glycero-3-phosphocholine (POPC) on SiO_2_; and (iii) monolayers made from POEPC on hydrophobic alkanethiolate substrates. The group determined the surface capacitance during bilayer formation and the response (amplitude) of voltage signal with respect to time. To calculate the quality of the mono- and bi-layers, the capacitance of the lipid films can be anticipated from the measured change in capacitance as shown in Figure 18a,b. This gives 2070 ± 40 nFcm^−2^ for the POEPC monolayer and 1290 ± 220 and 1160 ± 300 nFcm^−2^ for the POEPC and POPC bilayers, respectively. They noted a clear difference between POEPC and POEPC bilayer formation. For POEPC, the rapid decrease in capacitance starts directly after the vesicles reach the sensor surface, which takes only 3 min as shown in Figure 18d. For POPC, the response was instead delayed by 2–4 min. During this lag phase, there is only a slight decrease in capacitance. In terms of amplitude (peak voltage), they concluded that for POEPC on octadecanethiol (ODT), the peak voltage increases dramatically during the monolayer formation. For POEPC and POPC on SiO_2_, the situation is completely different. Here, the peak voltage first decreases rapidly during the initial stage of bilayer formation, and then it is increased. EIS studies also proved that this initial decrease is neither due to lipid monolayer formation nor capacitance response but is caused by the thin layer of conducting solvent in between the SiO_2_ and the bilayer. The lowest measured capacitance for POEPC and POPC bilayers in this study was 1070 and 990 nFcm^−2^, respectively.

**Figure 19:**
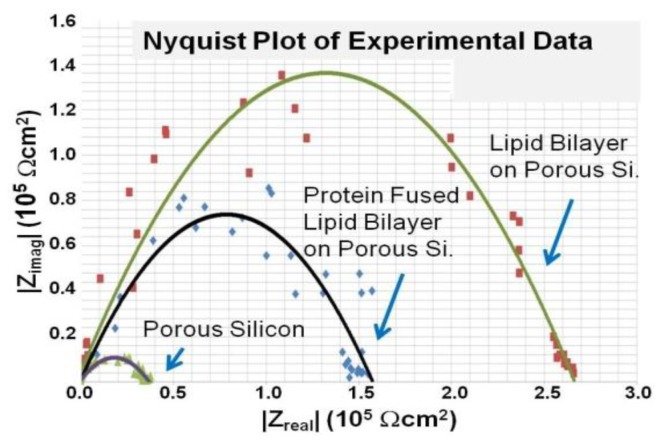
Nyquist plot with frequency range 0.1–100 kHz of the complete system. Reprinted with permission from Nano Science and Technology Institute [48].

Tantawi *et al*. [48,159] also performed EIS measurements on porous silicon supported lipid membrane fused with transmembrane protein. In this experiment, a bilayer of 1,2-dipalmitoyl-sn-glycero-3-phosphoethanolamine, 1,2-dipalmitoyl-sn-glycero-3-phosphoserine fused with epithelial sodium channel (ENaC) protein was suspended over their porous silicon membrane and electrically measured across the liquid filled vias. A Nyquist plot of the real and imaginary impedance of impedance from their experiment is presented in Figure 19. It is important to note that the impedance across the porous membrane is significantly smaller than the supported lipid bilayer and the membrane fused bilayer. Furthermore, measurements show that the impedance of the native bilayer is significantly larger than that of the membrane fused with the sodium channel.

The most important result of Tantawi’s *et al.* research [48,49,159] is the match of this data to biological measurements for both ENaC and the lipid bilayer. The lipid bilayer membrane showed a capacitance of approximately 0.63 μFcm^−2^, and a resistance of about 4 kΩcm^2^. Romer *et al*. [25], Hillebrandt *et al*. [80], Zhu *et al*. [155], Awayda *et al*. [181], Berdiev *et al*. [182] and Naumowicz *et al*. [183–185] recorded the membrane’s capacitance of about 0.67–0.95 μFcm^−2^ with varied compositions. Furthermore, Tantawi and his team also measured the capacitance of ENaC channels to be 0.57 μFcm^−2^ over six independent membrane measurements. Calculated capacitance matches closely with the EIS patch clamp data reported on ENaC using live frog oocyte cells by Awayda *et al*. [181], Berdiev *et al*. [182] and Hillebrandt, H. *et al*. [80]. Awayda reported a normalized capacitance of 0.33 μFcm^−2^ for ENaC fused into a BLM of the the same phospholipid bilayer composition used by Tantawi *et al*.

Table 1 provides an overview of published EIS data from different research groups. One quickly notes that the membrane’s capacitance and resistance values vary among different research efforts. The reason for these differences is the approach of EIS under different physiological conditions. For instance, different lipids, varying deposition methods and ratea, surface patterned below the lipid bilayer, the type of bilayer application technique, fabrication method of nanopores and their size, and input signal/voltage to electrodes are the important factors that drastically vary the EIS data. The majority of published work is focused on Gramicidin (A, B, C, D) due to its long term stability and ease of membrane fusion. The capacitance values published for various Gramicidin vary by an order of magnitude based on the conformity of the protein (its mutant form A, B, C or D) which is dictated by the temperature, the surface pressure of the lipid bilayer, the length of the fatty acid chains used, and most importantly, the static forces applied to the protein by the artificial template material patterned under the bilayer. To conclude, EIS gives in depth information about mechanical stability and the life time of membrane. In addition, it provides excellent *in vivo* mechanisms to record single and multiple ion movements across the membrane incorporated with different proteins.

As per Table 1, where,

DOPC: 1,2-di-sn-glycero-3-phosphocholine,
TR-DHPE: Dihexadecanoyl-phosphoethanolamine,
DPPE: 1,2-dipalmitoyl-sn-glycero-3-phosphoethanolamine,
DPPTE: 1,2-dipalmitoyl-sn-glycero-3-phosphoethanol,
DPPS: 1,2-dipalmitoyl-sn-glycero-3-phosphoserine,
DPHPC: 1,2-diphytanoyl-sn-glycero-3-phosphocholin,
DMPC: dimyristoylphosphatidylcholine,
DHADAB: dihexadecyldimethylammonium bromide,
POPG: 1-Palmitoyl-2-oleoyl-sn-glycero-3-[phosphor-rac-(1-glycerol)],
POEPC: 1-palmitoyl-2-oleoyl-sn-glycero-3-ethylphosphocholine and
POPC: 1-palmitoyl-2-oleoyl-sn-glycero-3-phosphocholine.

## 6. Conclusion and Future Directions

**Figure 20:**
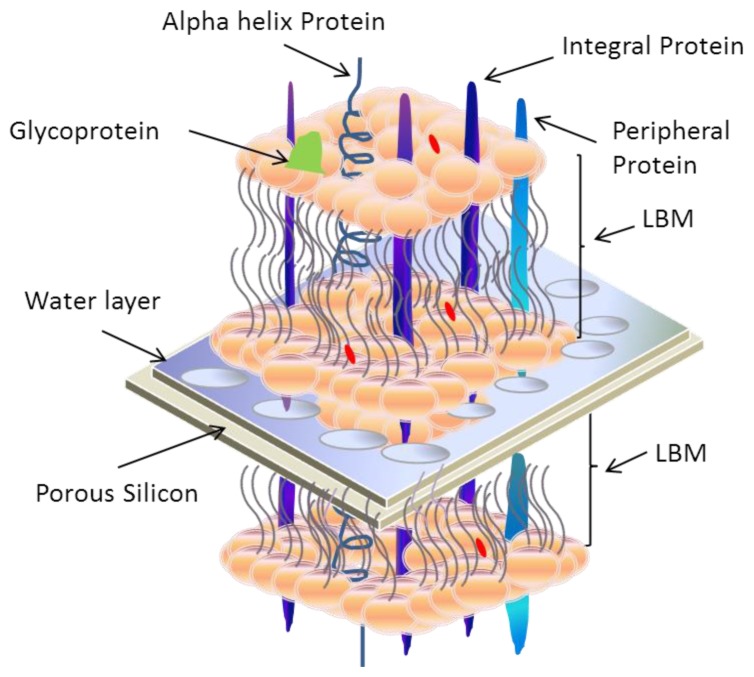
Prototype description of real time *in vivo* monitoring of different proteins fused in lipid bilayer membrane (LBM) on each side of porous silicon membrane.

Different reviews on artificial biological membranes have been published so far, but this review focuses exclusively on engineering lipid bilayer membranes (LBMs) incorporated with transmembrane proteins. It highlights the detailed structure of biological membrane, protein-protein interactions on surfaces and different parameters affecting physiological properties of proteins fused in supported LBMs. Among different supporting materials, this review revealed that porous silicon (PSi) is a promising material used as support to study proteins incorporated in artificial biological membranes. Electrochemical impedance spectroscopy (EIS) data measured by different research groups concludes that their experimental approaches will help to better understand the *in vivo* properties of cellular wall structure and record ion channels movements across the PSi supported membrane fused with transmembrane proteins. Currently, the authors of this review are studying the cell membranes of *E. coli* and mitochondria [186] using a new approach by spreading LBMs fused with different proteins on both sides of PSi. A summary of this work is presented in Figure 20. In this way, functional ions can easily pass through PSi supported membrane and may allow biologists to study proteins in a simulated environment.

## Figures and Tables

**Table 1:** Membrane’s capacitance and resistance using EIS published in recent years.

Reference	Supporting surface	Pore size (nm)	Lipid membrane	Protein	Membrane capacitance (μFcm^−2^)	Membrane resistance (GΩcm^2^)
[25]	Porous alumina	55–280	Bilayer (DPPTE and DPHPC)	Gramicidin (A, B, C, D)	0.65 ± 0.2	0.16
[155]	Porous silicon nitride	200–700	Bilayer (DOPC)	Gramicidin (A, B, C, D)	5.9	38.8–53.6 *
[80]	Glass slides coated with Indium-Tin-Oxide	-	Composed of 42 mol % DMPC, 49 mol % cholesterol, and 9 mol % DHADAB on regenerated cellulose by vesicle fusion	Gramicidin D	0.57	44 × 10^−5^
[150]	Porous alumina	200	Bilayer (DOPC and TR-DHPE)	α-hemolysin	-	9.0
[152]	Teflon with gold coated	0.02	Phosphatidylcholines	Gramicidin D & Proteoliposome	(1.8 ± 0.2) × 10^−3^	25.9 ± 4.1
[48,159]	Porous silicon	0.0002–0.002	Bilayer (DPPS and DPPE)	ENaC	0.63	4 × 10^−6^*
[179]	Alkanethiol (gold)	-	Monolayer (POEPC)	Gramicidin D	2.1 ± 0.04	-
	SiO_2_	-	Bilayer (POEPC and POPC)		1.29 ± 0.2	-
					1.16 ± 0.3	-
			Bilayer (POPG)	-	2.4 (ordered)	-
[180]	Porous alumina (ordered/non ordered)	20–50	Bilayer (DPPTE and DOPC)	-	0.99 (non-ordered with gold covered alumina), 0.90 (non-ordered)	4 × 10^−4^*

* Measured in GΩ only. Complete unit for membrane’s resistance was not provided.
